# Professor Renzi’s Account Af an Epidemic of Typhus, Accompanied with Appoplectic and Tetanic Symptoms, &c. &c.

**Published:** 1842-05-28

**Authors:** 


*An account of an Epidemic of Typhus, accompanied with Apoplectic and Tetanic symptoms, which was observed in the district of Cervaro, and parts adjacent, during the winter and commencement of the spring of* 1840. By Professor de Renzi.—This is one of several papers by different physicians on an epidemic which appears to have excited considerable attention. It broke out at Mignano in February, 1840, and caused much alarm from a report which spread among the vulgar that the cholera had made its appearance. This was at once ascertained to be false, and at the end of a month the disease ceased in Mignano, but began to spread at Cervaro and in other communes of that district. Almost all of the inhabitants were more or less affected by the epidemic influence, and suffered from giddiness, lassitude, and great depression of spirits; symptoms which were especially severe in those who had been attacked by intermittents during the previous year.

The disease made its onset differently in different cases. In some persons the first symptom was a sense of formication beginning at the feet and extending over the whole body; others suffered from general uneasiness, pain in the head or back, particularly in the cervical and dorsal regions, attended with difficulty on stooping forward or bending the neck. Occasionally this sensation extended to the lower limbs, and was accompanied with spasmodic contractions. Sometimes the disease was ushered in by an apoplectic seizure with loss of speech and consciousness, lasting for some hours, and followed by a kind of febrile reaction. Other persons fell down in convulsions, with trismus, the neck being drawn forcibly backwards, the whole trunk rigid, spasm of the extremities, and efforts to vomit, sometimes without anything being rejected, while at other times the patient would throw up some lumbrici.

With the exception of a few very mild cases, the disease ran much the same course, whatever were the symptoms which ushered in the attack. It might usually be divided into three stages: of which the first was characterized by violent irritation of the nervous centres; the second was the stage of reaction with gastro-nervous fever; the third was attended with putrid or adynamic symptoms. No particular crisis was observed, nor any discharge which could be looked on as critical.

The stage of reaction was accompanied with fever, a hard, quick, and frequent pulse, cephalagia, and a painful sense of retraction of the head. The pain in the head increased in severity, affecting principally the occiput and extending to the neck and along the spine. In some instances the pain in the spine was dreadfully severe, and the sacrum was referred to as the seat of the greatest suffering. When very violent, this pain was followed by opisthotonos, so complete as to bend the spine into the form of a roman S. Trismus, difficult deglutition, with disinclination for all drinks, subsultus, and a tremulous state of the limbs existed in the severest cases. The tongue was dry, the teeth were coated with sordes, the patient could scarcely speak, the voice was stridulous, except in those instances where there existed complete aphonia. The bowels were costive, and lumbrici were voided by stool, or crept out of the mouth. When the symptoms, however, presented such severity, the patients usually died in twenty-four or forty-eight hours; or if they survived they continued hemiplegic, or lost the use of some of their senses, or fell into a state of low, nervous fever.

In less formidable cases occasional remissions took place, and the nervous symptoms were not so severe, but even then a degree of tetanus and pain in the joints invariably existed. In these cases delirium came on about the fifth day, and was sometimes violent, at other times quiet and muttering. To the delirium coma succeeded, usually about the seventh day; but when the disease had a fatal issue coma set in very early, even without previous delirium, or after it had existed only for a few hours. When the patients lived until the second week, the prevailing symptoms were those of adynamic fever, and livid spots frequently appeared on the surface of the body.

General congestion of the intestinal canal, with softening of the mucous membrane of the colon, and injection of the vessels of the peritoneum were found after death. The bowels often contained lumbrici, and the spleen and liver were usually very large. The heart and large vessels contained black and liquid blood; the thoracic viscera generally were much congested, as were the vessels of the brain, but extravasations of blood into the cerebral substance were never met with.

The disease seems to have been very fatal; but Professor Renzi is unable to give statistical details concerning all the communes in which it prevailed. He states, however, that of 218 who were attacked, 116, or more than one-half, died. Those who principally fell victims to the epidemic were young men from sixteen to thirty, especially the robust and healthy. The duration of the disease was very various, some who were attacked dying within a few hours, others in the course of a week, some during the second or third week, and one even so late as fifty days after the seizure. As the epidemic declined, it by degrees assumed the characters of a gastro-rheumatic fever.

Oleaginous purgatives, small doses of calomel, and the solutions of acetate of ammonia and of tartar emetic, were the chief internal remedies in the milder cases; and warm baths, blisters to the extremities, and frictions with belladonna to the spine, the principal external applications. The severer cases were not benefited by any kind of treatment whatever.

Dr. Renzi attributes the disease to the injurious influence exerted on the atmosphere by the cultivation of rice, for though the disease appeared to spread by contagion, yet it did not present such violent symptoms anywhere else as in the immediate vicinity of the rice plantations.—*Il Filiatre Sebezio. Luglio*, 1840.

In the same Journal for May, 1841, is a description by Professor Renzi, of a similar epidemic which occurred at S. Marzano, and which, like the epidemic of Mignano, proved very fatal, fifty persons dying out of eighty who were attacked by it. The symptoms so closely resemble those observed at Mignano, that their detail is unnecessary, but the circumstances under which the disease broke out are worth notice. S. Marzano is seated on a level near the Saono, and all the streams from the neighbouring mountains traverse the surrounding country. In January, 1840, the Saono was so swollen by the rains that it overflowed its banks, and, inundating the plain, left the commune of S. Marzano in a sort of island. On the cessation of the rains unusually warm weather set in. The waters rapidly subsided, and the exhalations from the ground became exceedingly offensive. The peasantry, who were predisposed to disease by the privations they had undergone, suffered much from pursuing their labours in this poisonous air, and accordingly in January the disease broke out among them.

The number for September, 1840, contains an account by Dr. Salvatori of a kindred disease, the typhoid cephalagia, which was observed among the galley slaves at Procida. This paper affords proof of the contagious nature of the disease, thus confirming the opinion expressed by Professor Renzi. It also shows that when patients are not exposed to the evils of a malarious atmosphere, the disease loses much of its fatal character. At the commencement of April, 1840, some new convicts came to the galleys at Procida, having recently been discharged from the prisons at Potenza, where a disease was then prevailing exactly resembling the epidemic at Mignano. These were the first attacked; those convicts who had been longest employed in the galleys, or such as were inmates of the hospital for some chronic malady, suffered next. Other diseases then existing in the hospital soon put on the characters of this typhus, but it did not spread beyond the convict hospital and the galleys. Severe pains in the head and loins occurred as in the epidemic at Mignano. Trismus and coma were not invariably met with, and the whole course of the disease was so mild, that of sixty-two who were attacked by it only one died.

This epidemic in the *circondario* of Cervaro has been made the subject of a special work, as well as of several essays in the Italian Journals. Dr. Spada, who was appointed by the Neapolitan government to superintend the treatment of the disease, has written a book upon it, a brief notice of which appears in the Filiatre-Sebezio for December, 1840. This notice contains but little account of the malady beyond what we have already given. It is however interesting, inasmuch as it points out some analogies between the disease and some of those epidemics which a few centuries ago were prevalent throughout Europe. Dr. Spada suggests that there is a similarity between the fever and the acute scurvy of the middle ages, while many of its symptoms resemble those of a convulsive disease to which sheep are subject from eating the poisonous anemone. The reviewer remarks, that its convulsive symptoms bear a close resemblance to those of ergotism, but does not infer from this that the two diseases are alike in character. He labours indeed on the contrary to prove that the disease which prevailed at Cervaro was nothing else than an epidemic typhus fever. In this opinion we think most will acquiesce who are at all familiar with the descriptions of the malignant fevers which devastated Italy during the 15th, 17th, and early part of the 18th century.—*Brit. and For. Med. Rev.*

